# Multiple machine learning models for predicting major adverse cardiovascular events in dialysis with clinical and echocardiographic parameters: a retrospective cohort study

**DOI:** 10.1080/07853890.2026.2698905

**Published:** 2026-07-10

**Authors:** Mei Jin, Zikang Lin, Lingxiang Ma, Bing Li, Xuning Huang, Meihua Chen

**Affiliations:** Department of Ultrasound Medicine, Second Affiliated Hospital of Hainan Medical University, Haikou, Hainan, P.R. China

**Keywords:** Dialysis, major adverse cardiovascular events, machine learning, left ventricular global longitudinal strain, SHAP

## Abstract

**Background:**

Patients undergoing dialysis are at an elevated risk of cardiovascular events. This study aimed to develop machine learning (ML) prediction models to identify risk factors for major adverse cardiovascular events (MACE) in dialysis patients.

**Materials and Methods:**

This retrospective study included 203 patients undergoing dialysis with a median age of 45.0 years and 64.0% male. The participants were divided into training and test sets in a 7:3 ratio. LASSO regression selected characteristic variables from patients’general information, laboratory tests, and echocardiographic parameters (including global longitudinal strain [GLS]). Eight ML models were constructed,and SHAP analysis evaluated feature importance.

**Results:**

The incidence of MACE (including myocardial infarction, unstable angina, heart failure, and cardiovascular death) in dialysis patients was 38.92%. The average follow-up period was 18 months. LASSO regression identified eight feature variables. Among the ML models, AdaBoost demonstrated superior performance, with an AUC of 0.883 (95% CI: 0.830–0.937), accuracy of 0.804, sensitivity of 0.864 and specificity of 0.762 in the training set, and an AUC of 0.809 (95% CI: 0.706–0.912), accuracy of 0.750, sensitivity of 0.90 and specificity of 0.675 in the test set. The SHAP analysis identified N-terminal pro-brain natriuretic peptide (NT-proBNP) level, estimated glomerular filtration rate (eGFR), GLS and age as the four most important features for predicting MACE in patients undergoing dialysis (mean absolute SHAP values: 0.199, 0.176, 0.096 and 0.091, respectively).

**Conclusion:**

Elevated NT-proBNP, advanced age, reduced eGFR and impaired GLS were independently associated with an increased risk of MACE in patients undergoing dialysis.

## Introduction

Chronic kidney disease (CKD) is projected to become the world’s fifth leading noncommunicable disease by 2040 [1]. Patients with end-stage renal disease (ESRD), a population with particularly high morbidity and mortality, face a markedly increased risk of cardiovascular disease [2–4]. Approximately half of ESRD patients experience major adverse cardiovascular events (MACE) [5]. Renal replacement therapy, primarily comprising dialysis (hemodialysis or peritoneal dialysis) and transplantation, is the mainstay of treatment for ESRD [6]. Currently, over 3.5 million people worldwide receive hemodialysis or peritoneal dialysis, and this number is expected to more than double by 2030 [7]. While dialysis effectively removes toxins and corrects fluid and electrolyte imbalances [8], mortality remains high in this population, largely due to cardiovascular complications [9,10]. Therefore, close monitoring of cardiac function in dialysis patients is crucial to mitigate cardiovascular risk.

Left ventricular ejection fraction (LVEF) estimates systolic function based on geometric assumptions of the ventricle but reflects only relative volume changes, not early myocardial dysfunction [11]. In contrast, speckle-tracking echocardiography measures myocardial deformation through strain analysis, serving as a useful tool for detecting early myocardial injury [12]. Studies indicate that left ventricular global longitudinal strain (GLS) is more sensitive than LVEF in identifying ventricular dysfunction and provides superior prognostic information [13]. For instance, GLS has been shown to achieve an area under the working characteristic curve (AUC) of 0.883 (sensitivity 88.5%, specificity 88.5%) for predicting clinical progression, whereas LVEF performed less precisely with an AUC of 0.654 (sensitivity 70.4%, specificity 54%) [14]. Furthermore, three-dimensional speckle tracking imaging (3D-STI) overcomes a key limitation of the two-dimensional speckle tracking imaging – its inability to track motion across planes – by assessing myocardial motion and strain parameters in three dimensions [15].

Timely identification of the risk of MACE in patients undergoing dialysis is crucial for implementing targeted interventions. However, most existing prediction models for this population are based on traditional logistic regression, which may not fully capture complex, nonlinear relationships among risk factors. Machine learning (ML) methods, capable of modeling such intricate interactions, have demonstrated superior potential in various disease prediction tasks [16,17]. Therefore, this study aimed to develop and internally evaluate ML models using routine clinical, laboratory and echocardiographic data (including 3D-STI parameters) to predict MACE in dialysis patients.

## Methods

### Study population

**Figure 1.**
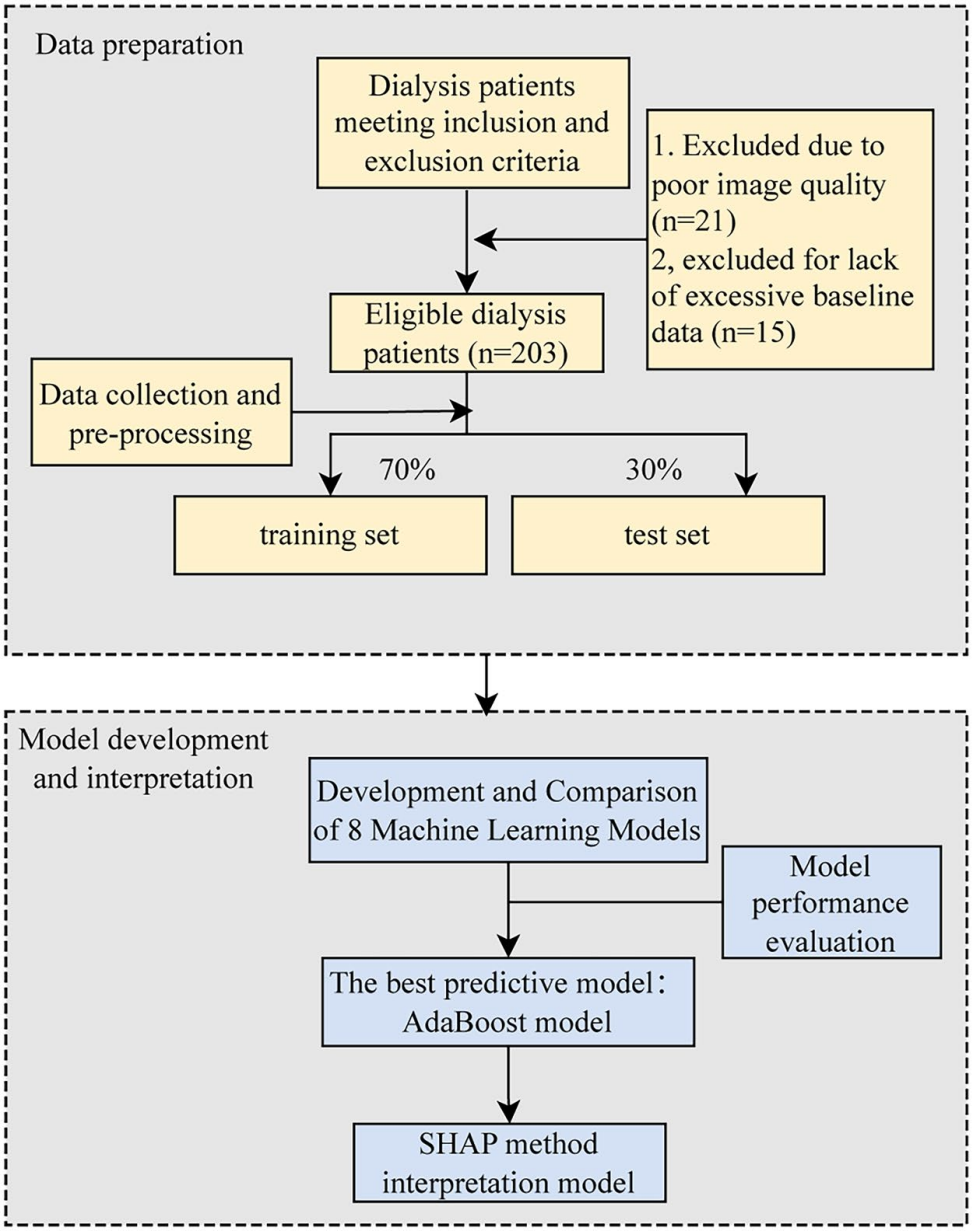
**Study flow diagram.** Patients who met the inclusion and exclusion criteria were divided into training sets and test sets according to 7:3. Eight machine learning models were trained, and the best model was selected based on evaluation indicators and parameter optimization. SHAP value was used to analyze the effect of features on major adverse cardiovascular events in dialysis patients.

This was a retrospective analytical study. The data between June 2021 and September 2025 was retrospectively analysed on February 2026. Dialysis patients who attended the Second Affiliated Hospital of Hainan Medical University between June 2021 and September 2025 were included. After applying the inclusion and exclusion criteria, 203 patients undergoing dialysis were included in the study. Inclusion criteria: According to the standards of the 2024 Clinical Practice Guidelines for the Evaluation and Management of Chronic Kidney Disease: patients diagnosed with stage 5 CKD, estimated glomerular filtration rate (eGFR) < 15 ml/(min·1.73 m^2^) [18]; patients receiving stable peritoneal dialysis and hemodialysis treatment under the diagnosis and treatment of clinicians. All patients required regular dialysis for more than 3 months. The exclusion criteria were as follows: (1) congenital heart disease, valvular heart disease, arrhythmia, and other diseases that cause myocardial involvement; (2) kidney transplant and (3) patients with poor image quality. The research methods are shown in Figure 1.

### Ethical statement

The study was approved by the Ethics Review Committee of the Second Affiliated Hospital of Hainan Medical University, and all participants signed informed consent forms (permit numbers:2026-K10-01). The study complied with the Declaration of Helsinki.

#### Demographic data

Demographic data were extracted from electronic medical records, including age, sex and body surface area (BSA), which was calculated using the Du Bois formula.

#### Clinical data

Clinical data comprised vital signs, laboratory parameters and comorbidities. Systolic and diastolic blood pressures were measured in millimeters of mercury (mmHg). The following laboratory parameters were measured: serum creatinine, blood urea nitrogen, N-terminal pro-brain natriuretic peptide (NT-proBNP), eGFR, hemoglobin, red blood cell count, serum calcium, serum phosphate, total cholesterol, triglycerides, low-density lipoprotein, high-density lipoprotein and parathyroid hormone. Comorbidities such as anemia, hypertension, diabetes and hyperparathyroidism were also documented.

#### Echocardiographic data acquisition

Transthoracic echocardiography was performed on all patients to assess cardiac structure and function, and to obtain the strain parameters for subsequent analysis. All examinations were conducted using a GE Vivid E95 ultrasonic diagnostic instrument, equipped with an M5Sc phased‑array probe (frequency range 1.4–4.6 MHz) for standard two‑dimensional imaging and a 4Vc three‑dimensional probe (frequency range 1.4–5.2 MHz) for volumetric acquisition. All acquired images were stored and analyzed offline using a GE EchoPac PC 203 workstation.

The participants were instructed to lie in the resting left lateral position for the echocardiographic examination. Using the M5Sc probe, the parasternal long-axis view of the left ventricle was acquired to measure the left atrial anteroposterior diameter (LAAP), interventricular septum end-diastolic thickness diameter (IVSTd), left ventricular posterior wall end-diastolic thickness diameter (LVPWTd), left ventricular end-diastolic diameter (LVEDd) and LVEF. The 4Vc probe was then used to acquire and store three consecutive cardiac cycles of apical four-chamber three-dimensional full-volume images, ensuring that the frame rate of the images exceeded 40% of the participant’s heart rate. By selecting 4D Auto LVQ and marking a point at the midpoint of the mitral annulus and a point at the apical endocardial surface, the software automatically traced the endocardial and epicardial curves, obtaining the GLS, global circumferential strain (GCS), global radial strain (GRS) and global area strain (GAS) of the left ventricle.

#### Outcomes

Patient follow-up was conducted *via* the hospital’s electronic medical record system and supplemented by telephone interviews. MACE was defined as the occurrence of myocardial infarction, unstable angina, heart failure or cardiovascular death. Cardiovascular death was confirmed based on medical records or detailed information obtained from family members during telephone interviews. It was defined as death caused by acute myocardial infarction, heart failure, sudden cardiac death or other vascular causes. The follow-up period spanned from patient enrollment until 30 October 2025. The average follow-up period was 18 months, and the median follow-up period was 12 months (interquartile range: 5–25 months).

### Statistical analysis

Normally distributed measures were expressed as mean ± standard deviation (x̅ ± s), non-normally distributed measures were expressed as median (Q1, Q3) and categorical data were expressed as frequencies and percentages [*n* (%)]. Intergroup comparisons of normally distributed measures were performed using independent samples *t*-tests. Comparisons between groups of non-normally distributed measures were performed using the Mann–Whitney U test. Comparisons between groups of categorical information were made using the χ 2 test. The overall percentage of missing data across the dataset was 2.8%. The variables with the highest missingness were N-terminal pro-brain natriuretic peptide (NT-proBNP, 9.8%) and serum phosphate (9.4%). Variables with missing values exceeding 20% of the total were excluded from the analysis. The remaining missing values were handled using multiple imputation. Of the remaining variables included in the study, 36% (specifically, 12 out of 33 variables) had missing values and were handled using multiple imputation.

A random sampling method was used to divide the subjects into training and internal test sets in a 7:3 ratio. The training set was used for hyperparameter optimization of the model, whereas the internal test set was used to internally evaluate the model performance. To prevent data leakage, variable selection was performed using the Least Absolute Shrinkage and Selection Operator (LASSO) regression exclusively within the training set. The optimal penalty coefficients (λ) were determined using a 10-fold cross-validation. LASSO regression enables feature selection by introducing an L1 regularization term that can force some regression coefficients to shrink to zero. We used eight ML algorithms, including random forest (RF), support vector machine (SVM), logistic regression (LR), decision tree (DTree), stochastic gradient descent (SGD), light gradient boost (LGB), adaptive boost (AdaBoost) and Bayes. The occurrence of MACE was used as the outcome variable, and other clinical metrics were used as independent variables. For each model, fivefold cross-validation was performed exclusively within the training set to optimize hyperparameters and assess the stability of model performance. The final model was then trained on the entire training set using the best-performing hyperparameters, and its AUC and other performance metrics were calculated. Finally, this finalized model was applied to the independent test set to generate predictions and calculate the test set AUC and other performance metrics. To evaluate the diagnostic performance of the models, we used several indicators, including the AUC, accuracy, specificity, sensitivity, and F1 score to comprehensively analyze the model efficacy, and evaluated the goodness-of-fit of each model based on the calibration curve. The clinical application value of each model was assessed using decision curve analysis (DCA).

We employ Shapley Additive Explanation (SHAP) for model interpretability analysis. This approach elucidates the output of a ML model through Shap values, which decompose model predictions into the contributions of individual features, thereby revealing complex nonlinear relationships. SHAP is utilized to assess the significance of both global and local features, as well as the dependencies and interactions among variables. Additionally, it facilitates the visualization of features for a given set of observations.

The adequacy of the sample size for multivariable modeling was assessed using the Events Per Variable (EPV) principle. With 79 recorded MACE events and 8 predictors included in the final models, the EPV was 9.9. Although slightly below the traditional threshold of 10, the use of LASSO regression ensured model stability and prevented overfitting through regularization.

The data processing and analysis in this study were conducted using Python 3.12.6. We primarily utilized the Numpy 1.26.4, Scipy 1.15.3, Pandas 2.3.2 and scikit-learn 1.7.1 libraries for the construction and evaluation of ML models. Visualization of the AdaBoost model was predominantly performed using the SHAP 0.48.0 library. Two-tailed P-values less than 0.05 were considered statistically significant.

## Results

### Comparison of variables between training set and test set

The study included 203 patients, of whom 79 experienced MACE. Participants were randomly allocated to the training and test sets in a 7:3 ratio, comprising 143 patients in the training set and 60 in the test set. Apart from body surface area, no statistically significant differences (*p* > 0.05) were observed between the training and test sets. Within the training set, individuals who experienced MACE demonstrated higher systolic blood pressure, LAAP, IVSTd, LVEDd, LVPWTd and N-terminal pro b-type natriuretic peptide (NT-proBNP), alongside lower eGFR and strain parameters compared to those without such events. Similarly, this difference was observed in eGFR and GLS between the groups with and without MACE in the test set (*p* < 0.05). See Tables 1 and 2.

**Table 1.** Comparison of variables between training and test sets.

Variables	Total (*n* = 203)	Training set (*n* = 143)	Test set (*n* = 60)	*P* value
MACE, *n* (%)				0.459
No	124 (61.1)	85 (59.4)	39 (65.0)	
Yes	79 (38.9)	58 (40.6)	21 (35.0)	
Dialysis method, *n* (%)				0.75
Peritoneal dialysis	105 (51.7)	75 (52.4)	30 (50.0)	
Hemodialysis	98 (48.3)	68 (47.6)	30 (50.0)	
Sex, *n* (%)				0.853
Female	73 (36.0)	52 (36.4)	21 (35.0)	
Male	130 (64.0)	91 (63.6)	39 (65.0)	
Age, years	45.0 (36.0, 56.0)	46.0 (37.0, 57.0)	43.0 (34.8, 52.2)	0.231
BSA, m²	1.6 ± 0.2	1.6 ± 0.1	1.7 ± 0.2	0.002
SBP, mm Hg	146.8 ± 22.8	147.0 ± 24.2	146.3 ± 19.0	0.846
DBP, mm Hg	89.2 ± 13.8	89.1 ± 13.9	89.5 ± 13.7	0.864
LAAP, mm	36.0 (32.0, 40.0)	37.0 (32.0, 40.0)	35.0 (31.8, 40.0)	0.56
IVSTd, mm	13.0 (12.0, 14.0)	13.0 (11.0, 14.0)	13.0 (12.0, 14.0)	0.141
LVEDd,mm	45.0 (42.0, 50.0)	45.0 (42.0, 50.0)	45.5 (42.0, 50.2)	0.394
LVPWTd,mm	12.0 (11.0, 14.0)	12.0 (11.0, 14.0)	13.0 (12.0, 14.0)	0.191
LVEF,%	64.0 (58.0, 68.0)	63.0 (58.0, 68.0)	65.0 (59.0, 68.0)	0.258
SCr, μmol/L	1055.0 ± 391.9	1049.4 ± 381.8	1068.3 ± 414.7	0.756
BUN, mmol/L	21.4 (17.3, 27.1)	22.3 (17.4, 27.6)	20.7 (17.1, 23.7)	0.114
NT-proBNP, pg/mL	6096.0 (1464.5, 14129.0)	5950.0 (1528.8, 14558.0)	6746.0 (1114.8, 13796.0)	0.882
eGFR, ml/(min*1.73m^2^)	12.0 (4.0, 15.0)	12.0 (4.0, 14.5)	12.5 (6.5, 17.2)	0.217
anemia, *n* (%)				0.317
No	45 (22.2)	29 (20.3)	16 (26.7)	
Yes	158 (77.8)	114 (79.7)	44 (73.3)	
Hb, g/L	107.3 ± 24.0	107.3 ± 23.9	107.3 ± 24.3	0.994
RBC,10^12^/L	3.8 ± 0.9	3.8 ± 0.8	3.9 ± 1.1	0.305
Calcium, mmol/L	2.3 (2.2, 2.4)	2.3 (2.2, 2.5)	2.3 (2.2, 2.4)	0.615
Phosphate, mmol/L	1.6 (1.3, 2.1)	1.6 (1.3, 2.2)	1.7 (1.3, 2.0)	0.749
Cholesterol, mmol/L	4.0 (3.2, 4.6)	4.0 (3.2, 4.7)	4.1 (3.5, 4.6)	0.512
triglycerides, mmol/L	1.1 (0.7, 1.5)	1.1 (0.7, 1.5)	1.2 (0.7, 1.7)	0.656
LDL, mmol/L	2.2 (1.8, 2.8)	2.2 (1.8, 2.9)	2.1 (1.7, 2.5)	0.609
HDL, mmol/L	1.1 (0.9, 1.3)	1.1 (0.9, 1.3)	1.1 (0.9, 1.2)	0.839
hyperparathyroidism, *n* (%)				0.929
No	60 (29.6)	42 (29.4)	18 (30.0)	
Yes	143 (70.4)	101 (70.6)	42 (70.0)	
Parathyroid hormone, pg/ml	271.9 (134.8, 444.7)	260.0 (123.8, 444.7)	274.8 (161.9, 431.0)	0.619
Duration of CKD, years	48.0 (24.0, 72.0)	48.0 (14.0, 62.4)	50.0 (24.0, 84.0)	0.049
Hypertension, *n* (%)				0.588
No	17 (8.4)	11 (7.7)	6 (10.0)	
Yes	186 (91.6)	132 (92.3)	54 (90.0)	
Diabetes, *n* (%)				0.627
No	165 (81.3)	115 (80.4)	50 (83.3)	
Yes	38 (18.7)	28 (19.6)	10 (16.7)	
GLS, %	−14.3 (−16.0, −12.5)	−14.2 (−16.0, −12.2)	−14.6 (−15.0, −12.9)	0.674
GCS, %	−15.0 (−17.0, −13.0)	−15.0 (−17.0, −12.0)	−14.9 (−17.0, −13.0)	0.722
GAS, %	−24.5 (−28.0, −21.0)	−24.4 (−28.0, −21.0)	−24.8 (−28.0, −21.9)	0.609
GRS, %	39.2 ± 11.9	38.7 ± 12.3	40.4 ± 11.0	0.366

MACE: major adverse cardiovascular events; BSA: body surface area; SBP: systolic blood pressure; SDP: diastolic blood pressure; LAAP: left atrial anteroposterior diameter; IVSTd: left ventricular interventricular septum end-diastolic thickness diameter; LVPWTd: left ventricular posterior wall end-diastolic thickness diameter; LVEDd: left ventricular end-diastolic diameter; LVEF: left ventricular ejection fraction; BUN: blood urea nitrogen; SCr: serum creatinine; eGFR: estimated glomerular filtration rate; NT-proBNP: N-terminal; pro-brain natriuretic peptide. Hb: hemoglobin; RBC: red blood cells; HDL: high-density lipoprotein; LDL: low-density lipoprotein; GLS: global longitudinal strain; GCS: global circumferential strain; GRS: global radial strain; GAS: global area strain; CKD: chronic kidney disease.

**Table 2.** Comparison of baseline data on whether major adverse cardiovascular events occurred between the training set and the test set.

	Training set (*n* = 143)			Test set (*n* = 60)		
Variables	Non-MACE (*n* = 85)	MACE (*n* = 58)	*P* value	Non-MACE (*n* = 39)	MACE (*n* = 21)	*P* value
Dialysis method, *n* (%)			0.065			0.417
Peritoneal dialysis	50 (58.8)	25 (43.1)		21 (53.8)	9 (42.9)	
Hemodialysis	35 (41.2)	33 (56.9)		18 (46.2)	12 (57.1)	
Sex, *n* (%)			0.148			0.712
Female	35 (41.2)	17 (29.3)		13 (33.3)	8 (38.1)	
Male	50 (58.8)	41 (70.7)		26 (66.7)	13 (61.9)	
Age, years	45.1 ± 12.3	49.2 ± 14.0	0.073	38.0 (32.0, 48.5)	49.0 (43.0, 63.0)	<0.001
BSA, m²	1.6 ± 0.2	1.6 ± 0.1	0.854	1.7 ± 0.2	1.6 ± 0.1	0.166
SBP, mm Hg	143.6 ± 23.5	151.9 ± 24.3	0.044	146.4 ± 18.5	146.2 ± 20.0	0.971
DBP, mm Hg	88.8 ± 13.7	89.6 ± 14.2	0.722	90.9 ± 13.4	86.9 ± 13.9	0.29
LAAP, mm	35.1 ± 6.6	38.7 ± 6.7	0.002	35.6 ± 6.2	36.6 ± 5.9	0.542
IVSTd, mm	12.0 (11.0, 13.0)	13.0 (12.0, 14.0)	0.002	13.0 (12.0, 14.0)	13.0 (12.0, 14.0)	0.643
LVEDd, mm	44.3 ± 6.2	47.9 ± 6.6	<0.001	47.1 ± 6.0	45.9 ± 5.4	0.477
LVPWTd, mm	12.0 (11.0, 13.0)	13.0 (12.0, 14.0)	0.012	13.0 (12.0, 14.0)	12.0 (12.0, 13.0)	0.584
LVEF, %	64.0 (58.0, 68.0)	62.0 (56.0, 67.0)	0.244	65.0 (60.0, 68.0)	65.0 (59.0, 68.0)	0.328
SCr, μmol/L	1035.7 ± 370.3	1069.5 ± 397.2	0.606	1074.6 ± 459.4	1056.5 ± 315.0	0.874
BUN,mmol/L	21.4 (17.3, 27.0)	23.1 (19.3, 29.2)	0.19	20.6 (17.2, 22.6)	20.8 (16.9, 24.8)	0.988
NT-proBNP, pg/mL	4287.0 (1117.0, 6746.0)	8885.0 (2691.0, 31960.5)	<0.001	6746.0 (1046.5, 7900.5)	8059.0 (1902.0, 19248.0)	0.348
eGFR, ml/(min*1.73m^2^)	13.0 (5.0, 15.0)	9.3 (4.0, 13.0)	0.042	13.0 (10.4, 18.5)	9.3 (3.0, 13.0)	0.015
anemia, *n* (%)			0.455			0.327
No	19 (22.4)	10 (17.2)		12 (30.8)	4 (19.0)	
Yes	66 (77.6)	48 (82.8)		27 (69.2)	17 (81.0)	
Hb, g/L	108.7 ± 23.7	105.3 ± 24.1	0.419	105.8 ± 23.4	110.3 ± 25.7	0.5
RBC,10^12^/L	3.9 ± 0.9	3.6 ± 0.8	0.076	3.8 (3.4, 4.3)	4.1 (3.3, 4.5)	0.852
Calcium, mmol/L	2.3 (2.2, 2.5)	2.3 (2.2, 2.4)	0.931	2.3 (2.2, 2.3)	2.3 (2.2, 2.5)	0.314
Phosphate, mmol/L	1.6 (1.3, 2.1)	1.6 (1.2, 2.4)	0.521	1.8 (1.3, 2.0)	1.7 (1.3, 1.9)	0.515
Cholesterol, mmol/L	4.1 ± 1.4	3.7 ± 1.2	0.082	4.1 (3.5, 4.6)	3.8 (3.5, 4.5)	0.525
triglycerides, mmol/L	1.1 (0.8, 1.7)	0.9 (0.7, 1.5)	0.174	1.0 (0.7, 1.4)	1.3 (1.0, 1.9)	0.109
LDL, mmol/L	2.4 (1.9, 3.0)	2.1 (1.7, 2.7)	0.074	2.3 (1.9, 2.6)	1.9 (1.6, 2.4)	0.046
HDL, mmol/L	1.2 (0.9, 1.3)	1.1 (0.9, 1.3)	0.261	1.1 (0.9, 1.4)	1.1 (0.9, 1.2)	0.438
hyperparathyroidism, n (%)			0.447			0.859
No	27 (31.8)	15 (25.9)		12 (30.8)	6 (28.6)	
Yes	58 (68.2)	43 (74.1)		27 (69.2)	15 (71.4)	
Parathyroid hormone,pg/ml	292.6 (153.4, 409.0)	250.8 (116.2, 495.5)	0.952	299.7 (182.2, 504.8)	221.4 (160.7, 397.1)	0.457
Duration of CKD,years	48.0 (24.0, 60.0)	43.5 (12.0, 84.0)	0.416	48.0 (24.0, 69.5)	65.0 (36.0, 120.0)	0.093
Hypertension, *n* (%)			0.21			1
No	9 (10.6)	2 (3.4)		4 (10.3)	2 (9.5)	
Yes	76 (89.4)	56 (96.6)		35 (89.7)	19 (90.5)	
Diabetes, *n* (%)			0.118			0.468
No	72 (84.7)	43 (74.1)		34 (87.2)	16 (76.2)	
Yes	13 (15.3)	15 (25.9)		5 (12.8)	5 (23.8)	
GLS, %	−15.2 (−18.0, −14.0)	−13.0 (−15.0, −11.0)	< 0.001	−14.9 (−17.0, −13.8)	−13.0 (−15.0, −12.0)	0.011
GCS, %	−15.0 (−18.0, −13.0)	−14.0 (−16.0, −11.0)	0.024	−14.9 (−17.0, −13.0)	−14.7 (−16.0, −12.0)	0.327
GAS, %	−25.2 ± 5.1	−22.5 ± 6.2	0.005	−25.4 ± 4.9	−23.7 ± 4.8	0.207
GRS, %	40.6 ± 10.8	36.0 ± 13.7	0.025	41.9 ± 11.3	37.6 ± 9.8	0.148

MACE: major adverse cardiovascular events; BSA: body surface area; SBP: systolic blood pressure; SDP: diastolic blood pressure; LAAP: left atrial anteroposterior diameter; IVSTd: left ventricular interventricular septum end-diastolic thickness diameter; LVPWTd: left ventricular posterior wall end-diastolic thickness diameter; LVEDd: left ventricular end-diastolic diameter; LVEF: left ventricular ejection fraction; BUN: blood urea nitrogen; SCr: serum creatinine; eGFR: estimated glomerular filtration rate; NT-proBNP: N-terminal; pro-brain natriuretic peptide. Hb: hemoglobin; RBC: red blood cells; HDL: high-density lipoprotein; LDL: low-density lipoprotein; GLS: global longitudinal strain; GCS: global circumferential strain; GRS: global radial strain; GAS: global area strain; CKD:Chronic kidney disease.

### Machine learning feature selection

**Figure 2.**
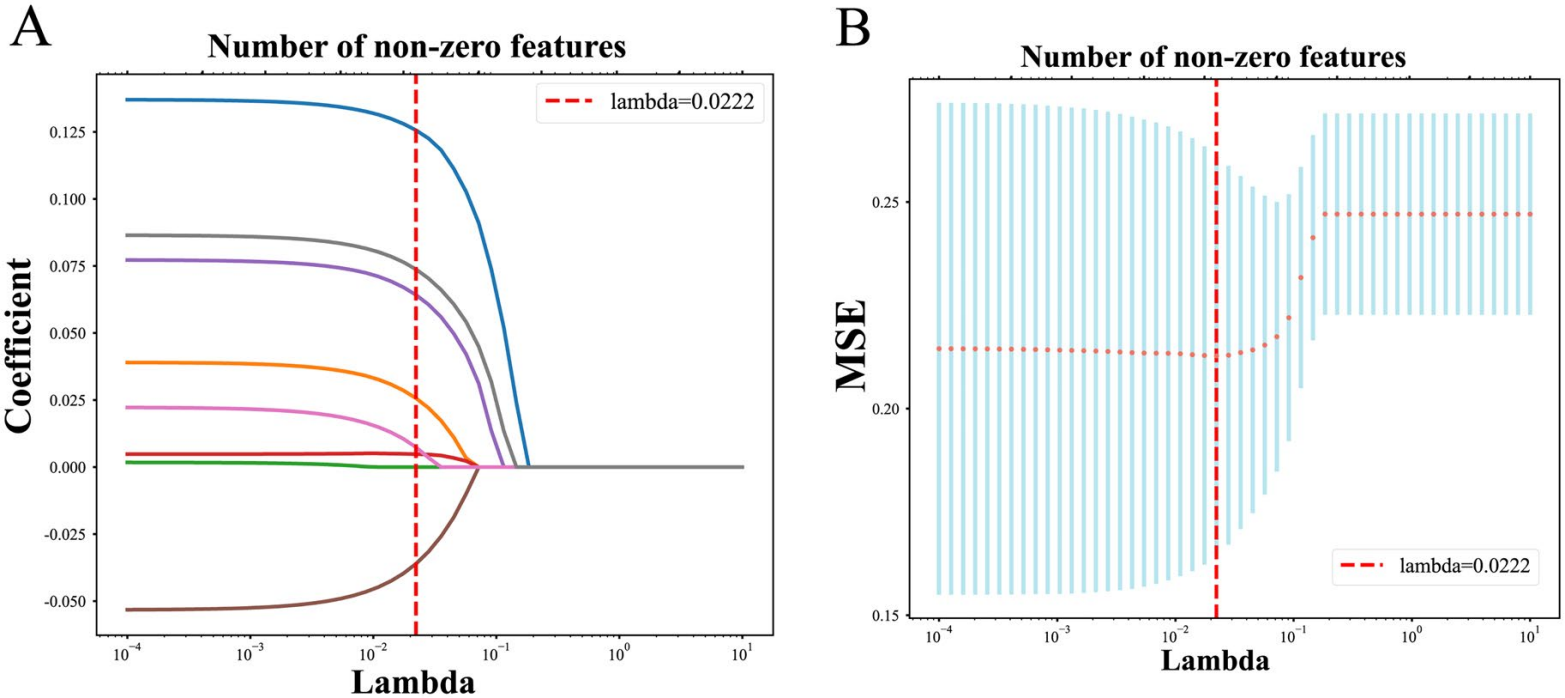
**Feature selection based on Lasso regression.** (A) The Lasso path diagram shows the correlation between the L1 norm and different coefficients in Lasso regression. (B) The Lasso cross-validation graph shown the correlation between λ and binomial deviation.

**Figure 3.**
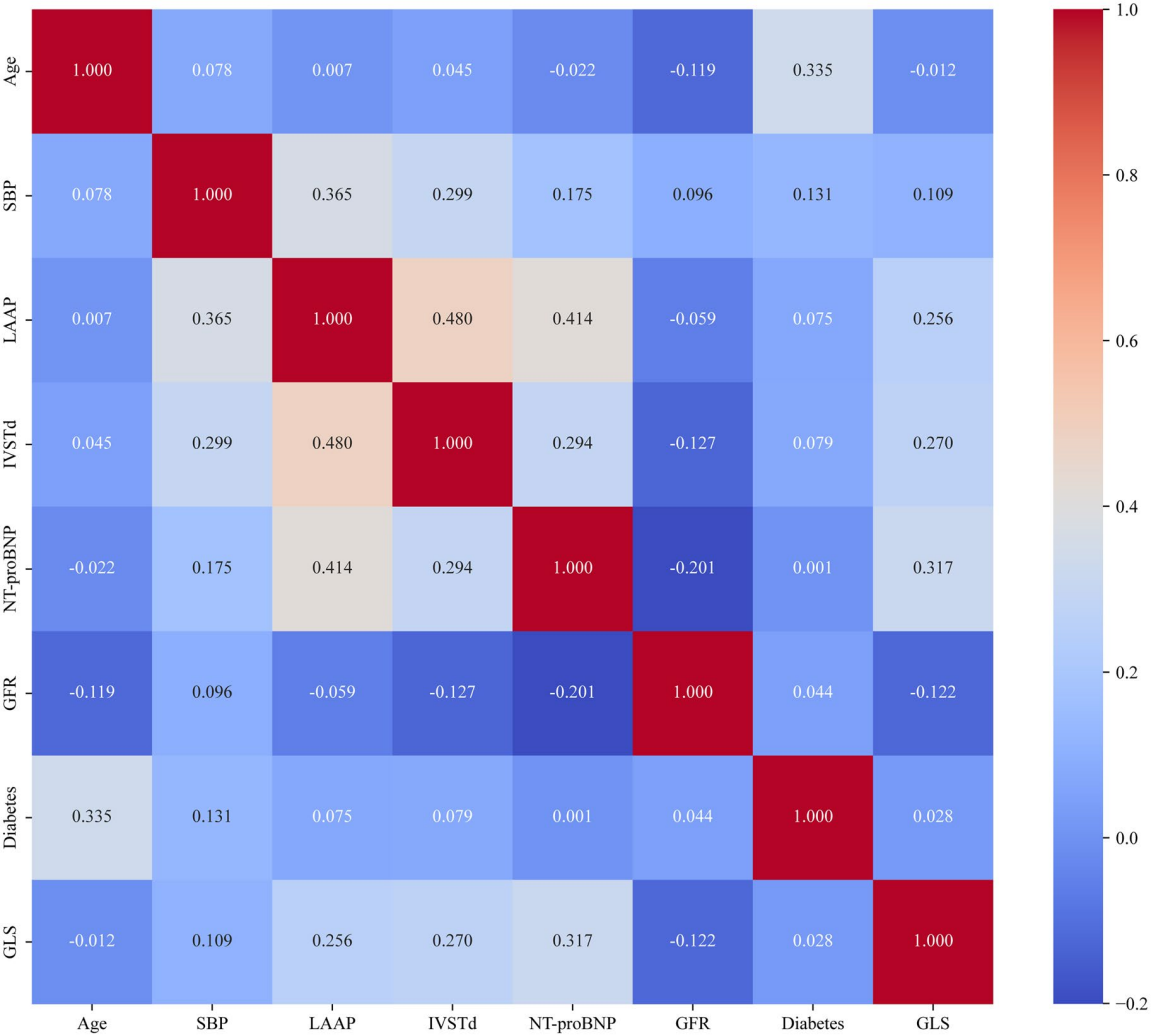
**Correlation heatmap.** Correlation heatmap shown correlations between the included characteristic variables.

The LASSO regression model incorporated 33 variables, with the optimal penalty coefficient λ determined through 10-fold cross-validation. Eight variables with nonzero coefficients were identified at the optimal λ (λ = 0.0222) as key predictors of MACE in dialysis patients, including age, systolic blood pressure, LAAP, IVSTd, NT-proBNP, eGFR, diabetes and GLS. The LASSO regression path diagram and cross-validation plot are shown in Figure 2. No significant correlations (*r* < 0.6) were detected among the variables (Figure 3).

### Model development and comparison

**Figure 4.**
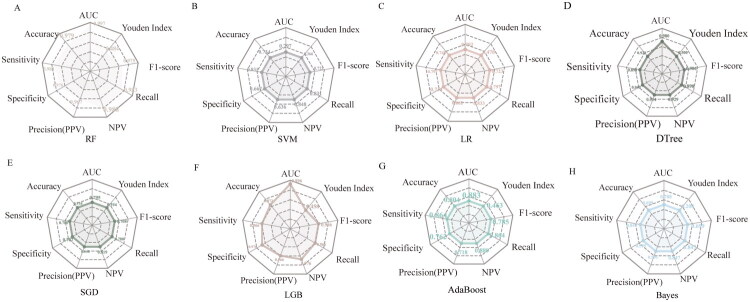
**Radar charts in the training set.** The radar charts show the performance of the eight machine learning algorithm models in the training set.

**Figure 5.**
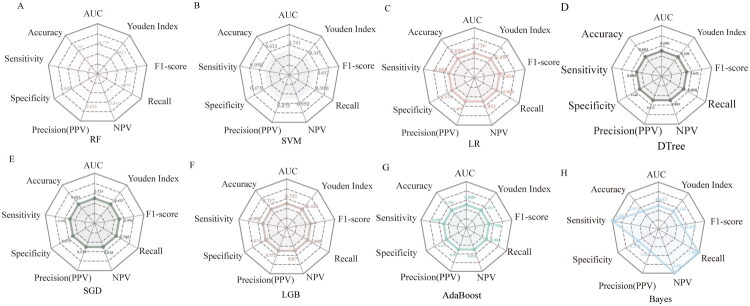
**Radar charts in the test set.** The radar charts show the performance of the eight machine learning algorithm models in the test set.

**Figure 6.**
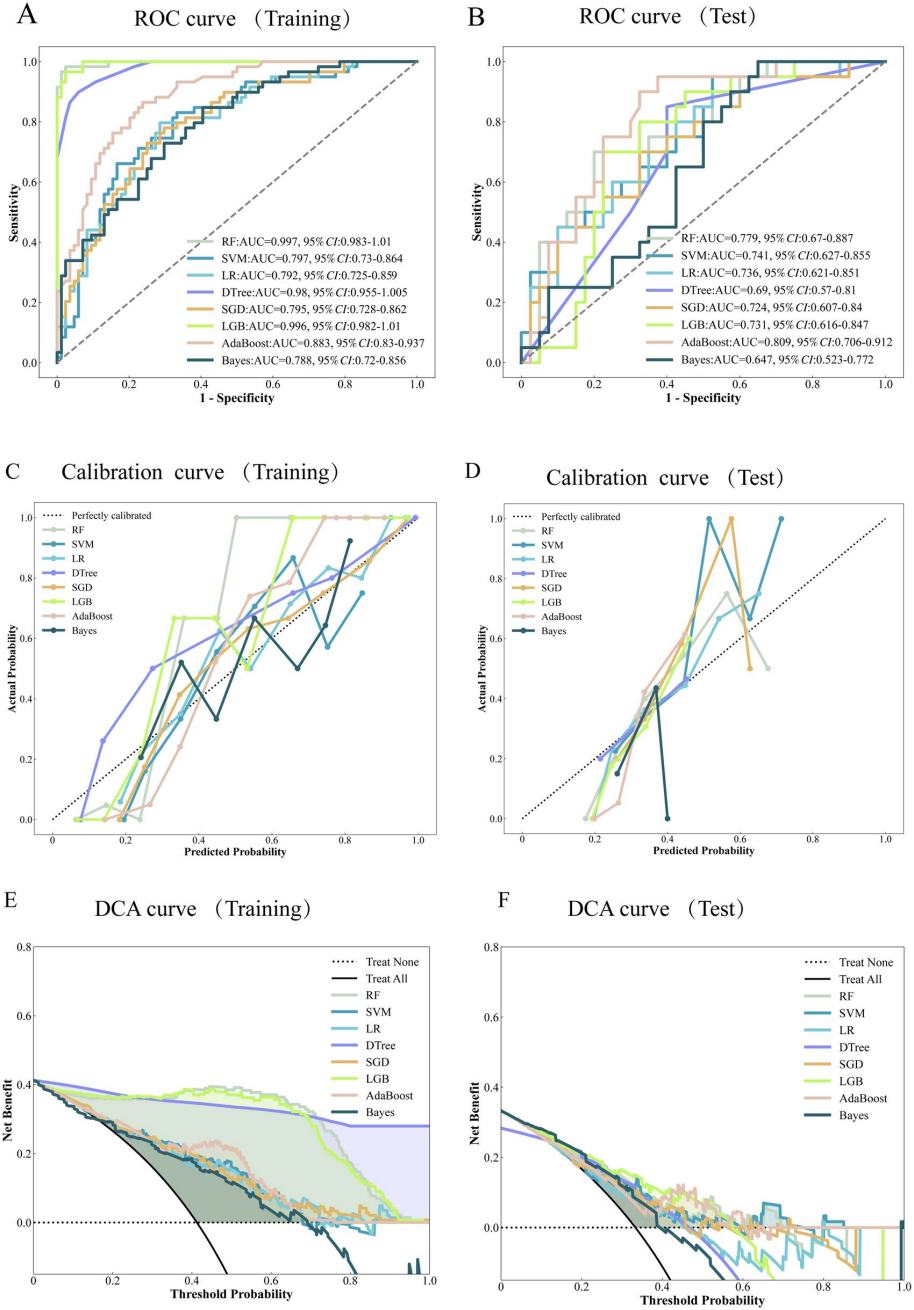
**Model performance comparison.** (A) ROC curves for different machine learning models in the training set and validation set predicted major adverse cardiovascular events. (B) Calibration curves for different models in the training set and test set. (C) DCA curves for different models in the training set and test set.

Following the standardization of the eight selected variables, eight ML algorithms were evaluated: RF, SVM, LR, DTree, SGD, LGB, AdaBoost and Bayesian methods. AdaBoost emerged as the most effective model for predicting MACE in dialysis patients. In the training set, the model achieved an area under the curve (AUC) of 0.883 (95% CI: 0.830–0.937), accuracy of 0.804 (95% CI: 0.738–0.870), sensitivity of 0.864 (95% CI: 0.807–0.922) and specificity of 0.762 (95% CI: 0.691–0.833). More importantly, in the internal test set, the model maintained robust performance, demonstrating an AUC of 0.809 (95% CI: 0.706–0.912), accuracy of 0.750 (95% CI: 0.637–0.863), sensitivity of 0.900 (95% CI: 0.820–0.980) and specificity of 0.675 (95% CI: 0.553–0.797). Although RF, DTree and LGB demonstrated high AUCs (0.997, 0.980 and 0.996, respectively), they exhibited significant overfitting in the test set. The performance of each model is detailed in Tables 3 and 4 and Figures 4 and 5. The ROC, DCA, and calibration curves for the training and internal test sets of each model are shown in Figure 6.

**Table 3.** Evaluation of the effectiveness of each model in the training set.

Model	AUC	Accuracy	Specificity	Sensitivity	F1-score	Precison
RF	0.997 (0.983–1.000)	0.979 (0.954–1.000)	0.976 (0.949–1.000)	0.983 (0.960–1.000)	0.975 (0.947–1.000)	0.967 (0.936–0.998)
SVM	0.797 (0.730–0.864)	0.734 (0.661–0.808)	0.667 (0.589–0.745)	0.831 (0.768–0.893)	0.721 (0.646–0.795)	0.636 (0.557–0.716)
LR	0.792 (0.725–0.859)	0.748 (0.676–0.820)	0.714 (0.639–0.789)	0.797 (0.730–0.864)	0.723 (0.649–0.797)	0.662 (0.584–0.740)
DTree	0.980 (0.955–1.000)	0.923 (0.878–0.968)	0.940 (0.900–0.981)	0.898 (0.848–0.949)	0.906 (0.857–0.955)	0.914 (0.867–0.961)
SGD	0.795 (0.728–0.862)	0.734 (0.661–0.808)	0.702 (0.627–0.778)	0.780 (0.711–0.849)	0.708 (0.632–0.783)	0.648 (0.569–0.727)
LGB	0.996 (0.982–1.000)	0.972 (0.943–1.000)	0.976 (0.949–1.000)	0.966 (0.935–0.997)	0.966 (0.935–0.997)	0.966 (0.935–0.997)
AdaBoost	0.883 (0.830–0.937)	0.804 (0.738–0.870)	0.762 (0.691–0.833)	0.864 (0.807–0.922)	0.785 (0.716–0.853)	0.718 (0.644–0.793)
Bayes	0.788 (0.720–0.856)	0.699 (0.623–0.775)	0.595 (0.514–0.677)	0.847 (0.788–0.907)	0.699 (0.623–0.775)	0.595 (0.514–0.677)

RF: Random forest; SVM: support vector machine; LR: logistic regression; DTree: decision tree; SGD: stochastic gradient descent; LGB: light gradient boost; AdaBoost: adaptive boost.

**Table 4.** Evaluation of the effectiveness of each model in the test set.

Model	AUC	Accuracy	Specificity	Sensitivity	F1-score	Precison
RF	0.779 (0.670–0.887)	0.767 (0.656–0.877)	0.800 (0.695–0.905)	0.700 (0.581–0.819)	0.667 (0.544–0.789)	0.636 (0.511–0.761)
SVM	0.741 (0.627–0.855)	0.633 (0.508–0.758)	0.475 (0.345–0.605)	0.950 (0.890–1.000)	0.633 (0.508–0.758)	0.475 (0.345–0.605)
LR	0.736 (0.621–0.851)	0.650 (0.526–0.774)	0.575 (0.447–0.703)	0.800 (0.695–0.905)	0.604 (0.477–0.731)	0.485 (0.355–0.614)
DTree	0.690 (0.570–0.810)	0.683 (0.562–0.804)	0.600 (0.473–0.727)	0.850 (0.756–0.944)	0.642 (0.517–0.766)	0.515 (0.386–0.645)
SGD	0.724 (0.607–0.840)	0.683 (0.562–0.804)	0.675 (0.553–0.797)	0.700 (0.581–0.819)	0.596 (0.468–0.723)	0.519 (0.389–0.648)
LGB	0.731 (0.616–0.847)	0.717 (0.599–0.834)	0.675 (0.553–0.797)	0.800 (0.695–0.905)	0.653 (0.529–0.777)	0.552 (0.423–0.681)
AdaBoost	0.809 (0.706–0.912)	0.750 (0.637–0.863)	0.675 (0.553–0.797)	0.900 (0.820–0.980)	0.706 (0.587–0.824)	0.581 (0.453–0.709)
Bayes	0.647 (0.523–0.772)	0.567 (0.438–0.695)	0.350 (0.226–0.474)	0.999 (0.977–1.000)	0.606 (0.479–0.733)	0.435 (0.306–0.563)

RF: Random forest; SVM: support vector machine; LR: logistic regression; DTree: decision tree; SGD: stochastic gradient descent; LGB: light gradient boost; AdaBoost: adaptive boost.

### SHAP analysis of best machine learning model predictions

**Figure 7.**
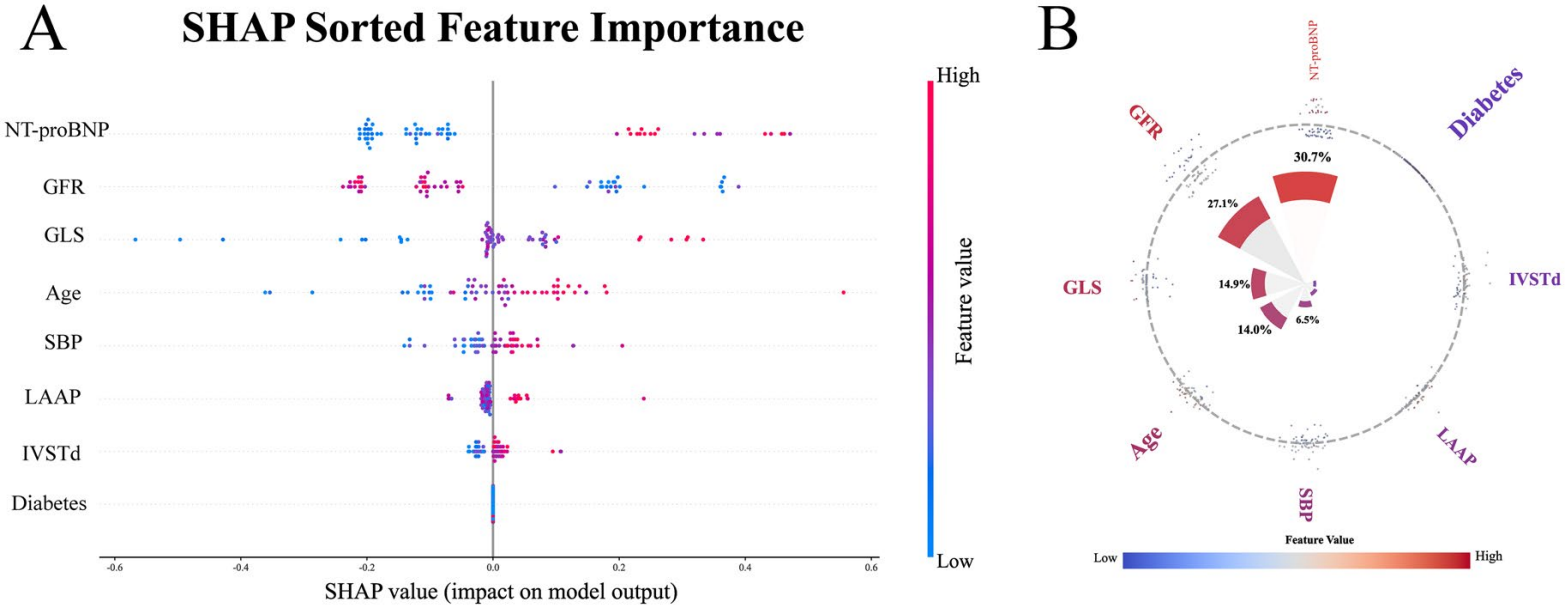
**Global model explanation by the SHAP method.** (A) The Shap hive diagram illustrating the distribution of SHAP values for each feature. The color gradient represents the feature values (red for high values and blue for low values). (B) Characteristic effect double ring diagram, the inner ring structure representing the feature importance distribution, and the radial distance of the outer ring scatter representing the SHAP value, and the farther away from the ring the ring, the greater the impact.

Given AdaBoost’s superior performance among the eight ML models, it was selected for model interpretation. The SHAP method was employed to rank the importance of feature variables, identifying NT-proBNP, eGFR, GLS and age as the four most influential features in the model predictions (mean absolute SHAP values: 0.199, 0.176, 0.096 and 0.091, respectively). SHAP hive plots and double-loop plots (see Figure 7) indicated that higher NT-proBNP levels and older age (red) were associated with an increased likelihood of MACE (right), whereas reduced eGFR and GLS were also correlated with a higher propensity for such events.

## Discussion

In this study, we developed ML models to predict MACE in dialysis patients. The aim of this study was to provide evidence for clinicians to assess the risk of MACE in dialysis patients. The main findings of this study are as follows: First, in identifying cardiac dysfunction in dialysis patients, GLS was a more sensitive indicator than the conventional LVEF. Second, among the eight ML models evaluated, the AdaBoost model demonstrated the best performance, with an AUC of 0.883 in the training set and 0.809 in the test set. Finally, to improve model interpretability, SHAP visual analysis was employed. The SHAP summary plot revealed that NT-proBNP level, eGFR, GLS, and age were the most influential predictors for MACE events in this dialysis cohort.

The development of ML technology has provided new methods and tools for predicting MACE in dialysis patients. Different ML models and algorithms have their own advantages. For example, LR is a powerful and mature supervised classification method that is well-suited for handling linear data [19]. However, the model’s accuracy is not high when there are complex relationships between the variables [16]. The SVM model is more robust than the LR model and is good at handling linear and nonlinear data [20]. However, the model is not suitable for handling large datasets with a large number of features or missing values. The RF model, on the other hand, is well suited to handle large datasets: High accuracy can be achieved by combining multiple decision trees. If the model overfits the noise in the training data, it performs poorly on new data [21]. DTree classifies data items by representing various judgment logics and corresponding results in a tree structure; however, the model is less stable [22]. The LGB Machine histogram-based splitting algorithm is fast to train but sensitive to hyperparameters [23]. Bayes is extremely fast to compute, almost no training time, suitable for high dimensional data but relies on independence assumptions [16]. The SGD is suitable for large-scale datasets, but its convergence is unstable and requires multiple iterations and early stops [24]. Among the eight models, AdaBoost exhibited the best predictive value. The AdaBoost algorithm, which is based on a weighted voting mechanism in ensemble learning, optimizes the model performance by iteratively updating the distribution of the training sample weights and demonstrates strong stability against outliers.

The SHAP method is derived from the Shapley value theory in game theory; its core is to quantify the contribution of each feature to the output of a ML model, clarifying how each feature influences the final prediction probability. The individual prediction results were presented as the sum of the base prediction value and SHAP values of all features [25]. Based on the SHAP values, the top four features in our study were NT-proBNP, eGFR, GLS and age. Consistent with previous studies [26–29], higher NT-proBNP levels and older age increased the model’s risk prediction, whereas higher eGFR and GLS reduced risk predictions.

Our study has pinpointed NT-proBNP as the paramount factor linked to MACE within our dialysis patient cohort. Elevated NT-proBNP levels are a recognized marker of cardiac dysfunction risk in cardiovascular disease, and this correlation is evident in dialysis populations as well. Irrespective of the dialysis method employed, whether hemodialysis or peritoneal dialysis, a heightened NT-proBNP level is indicative of an increased risk of cardiac dysfunction and serves as an independent predictor of both all-cause and cardiovascular mortality [26,30]. NT-proBNP is predominantly secreted by ventricular myocytes in response to wall stress, such as that caused by elevated cardiac filling pressures. In the context of chronic kidney disease, the prevalence of persistent volume overload and hypertension results in sustained ventricular wall tension, which in turn stimulates NT-proBNP production and release [31,32]. Consequently, vigilant monitoring of NT-proBNP levels is clinically justified, and strategies aimed at mitigating its underlying causes, such as volume overload, may contribute to reducing cardiovascular risk and mortality.

Our study findings also revealed a significant association between a lower eGFR and a higher incidence of MACE in dialysis patients. This underscores the importance of renal impairment severity as a determinant of cardiovascular risk, which is consistent with the findings of Grams and colleagues [29]. The cause of this phenomenon lies in the continuous decline in the glomerular filtration rate. As a result, the detoxification, excretion of water and regulation of endocrine activities progressively deteriorate. This leads to the accumulation of large amounts of uremic toxins, inflammatory factors and oxidative stress products in the body, which continuously damage vascular endothelial function and significantly increase the likelihood of cardiovascular diseases [33]. Additionally, reduced kidney function can trigger overactivation of the renin-angiotensin-aldosterone system, exacerbating water and sodium retention and excessive volume load. Over time, this places additional strain on the heart, leading to pathological changes, such as left ventricular hypertrophy and myocardial remodeling, ultimately increasing the risk of serious adverse cardiovascular events [34].

The study demonstrated that LVEF did not differ significantly between dialysis patients with and without MACE, whereas GLS exhibited a statistically significant difference between the two groups. This finding is consistent with prior evidence indicating that GLS serves as a more sensitive marker of subclinical myocardial dysfunction compared with LVEF [35]. Mechanistically, GLS quantifies the longitudinal deformation of the left ventricle, a motion predominantly governed by the contraction of the subendocardial longitudinal myocardial fibers. Given that the subendocardium is particularly vulnerable to ischemia, uremic toxin exposure and hemodynamic stress, GLS demonstrates high sensitivity to early myocardial injury, even when global systolic function, as reflected by LVEF, remains within the normal range [13]. In line with this pathophysiological rationale, our analysis further revealed that lower GLS values were independently associated with a higher risk of incident MACE in this population cohort. This observation aligns with previous studies that have established GLS as a robust prognostic indicator, retaining its predictive value even in patients with preserved LVEF [36,37]. Collectively, these results reinforce the clinical utility of 3D-STI derived parameters, particularly GLS, in the early detection of subtle contractile impairment, thereby advocating for their broader integration into routine cardiovascular monitoring for dialysis patients.

Age emerged as a robust predictor of MACE, reflecting the well-established accumulation of cardiovascular risk over time. With advancing age, individuals experience arterial stiffening, endothelial dysfunction and cumulative subclinical atherosclerosis – all of which synergistically elevate the likelihood of adverse cardiac events, particularly in the vulnerable dialysis population where accelerated biological ageing is prevalent [38]. Furthermore, our SHAP analysis identified LAAP, IVSTd, systolic blood pressure and diabetes as contributors to MACE risk. Increased IVSTd reflects concentric left ventricular remodelling, commonly driven by the dual burden of pressure overload and volume overload in ESKD. Over time, septal thickening progresses to myocardial fibrosis and diastolic dysfunction [39]. Evaluating left atrial size could offer further prognostic insights, given that left atrial enlargement is a recognized independent predictor of adverse cardiovascular outcomes [40]. The left atrium plays an important role in regulating left ventricular function, contributing to up to one-third of the cardiac output. For patients with chronic kidney disease, the left atrium is more sensitive to fluid overload and elevated left ventricular filling pressure, which may reduce the left atrium’s contribution to diastolic function and increase left atrial volume due to elevated left ventricular filling pressure [41]. In dialysis patients, elevated SBP is primarily driven by chronic volume overload and arterial stiffness, which increases left ventricular afterload and accelerate the progression of heart failure [42]. Diabetes exacerbates cardiovascular risk not only by accelerating systemic atherosclerosis but also by inducing diabetic cardiomyopathy, characterized by myocardial fibrosis and diastolic dysfunction [43]. These findings underscore the multifactorial nature of cardiovascular risk in dialysis patients, where cardiac mechanics, hemodynamic load, renal function and metabolic parameters collectively interact to precipitate adverse events.

## Limitations

This study had several limitations. Firstly, its single-center design may limit the generalizability of the model. Secondly, given the relatively small sample size and limited number of outcome events, there remains an inherent risk of overfitting, which may further constrain the model’s generalizability to broader populations. Although we mitigated this risk by employing LASSO regression for feature selection and utilizing cross-validation, this limitation cannot be entirely eliminated. Thirdly, the model lacks external validation, which is essential to confirm its robustness and clinical applicability. Finally, due to a 4-year enrollment period (June 2021–September 2025) and a unified cutoff (October 2025), later-enrolled patients had shorter follow-up. This imbalance may underestimate MACE incidence in these patients. Therefore, validation in larger, multicenter, prospective cohorts with uniform and sufficient follow-up periods is necessitated for future clinical application.

## Conclusion

In summary, we developed a machine-learning model based on routine clinical and echocardiographic data to predict MACE in patients undergoing dialysis. Using SHAP analysis, the model not only identified but also quantified the directional impact of key predictors, demonstrating that elevated NT-proBNP and older age increased MACE risk, while higher eGFR and better GLS were protective. Pending future external validation, this predictive model should be construed as a preliminary tool that has the potential to support risk stratification efforts in dialysis patients.

## Data Availability

The data analyzed during the current study is available from the corresponding author on reasonable request.

## Data availability statement

The data that support the findings of this study are not publicly available due to privacy reasons but are available from the corresponding author upon reasonable request.
